# Supplementary material for: Evaluation of Prolonged Walking in Persons with Multiple Sclerosis: Reliability of the Spatio-Temporal Walking Variables during the 6-Minute Walk Test
Source: Sensors (Basel). 2021 Apr 28;21(9):3075. doi: 10.3390/s21093075 (PMC8125198; doi:10.3390/s21093075)
Supplement: Supplementary file 1 [file sensors-21-03075-s001.zip › sensors-1162810-supplementary.pdf]

Supplementary data 1: Definitions of the measurements of spatio-temporal variables

From ProtoKinetics LLC. ProtoKinetics Movement Analysis Software: Measurements and Definitions, Version 5.09C2. July 2018

Velocity: Is the result of dividing the sum of all the measurements of stride length by the sum of all the stride time of both feet; expressed in meters per second (m/s).

Cadence: It is defined as the number of footfalls minus one, divided by the ambulatory time, and converted to minutes (steps/min).

Stride Length: The distance from the heel of one foot to the following heel of the same foot (m) and takes into account the instantaneous direction of progression as outlined by Huxham et al. (Defining spatial parameters for non-linear walking. Gait Posture. 2006;23(2):159-63). The unit of measure is meter.

Stride Width: It is the perpendicular distance between the line connecting the two ipsilateral foot heel contacts (stride) with the contralateral heel between those events. The unit of this measurement is meter.

Total double support time: The sum of all periods when both feet are in contact with the ground during stance phase. The two periods when both feet are on the floor, are called initial double support and terminal double support. Initial double support occurs from heel contact of one footfall to toe-off of the opposite footfall. Terminal double support occurs when the opposite footfall heel strike to support the other's footfall toe-off. Total double support is the sum of the initial double support added to the terminal double support; this was expressed as a percentage of the gait cycle time for the same foot.

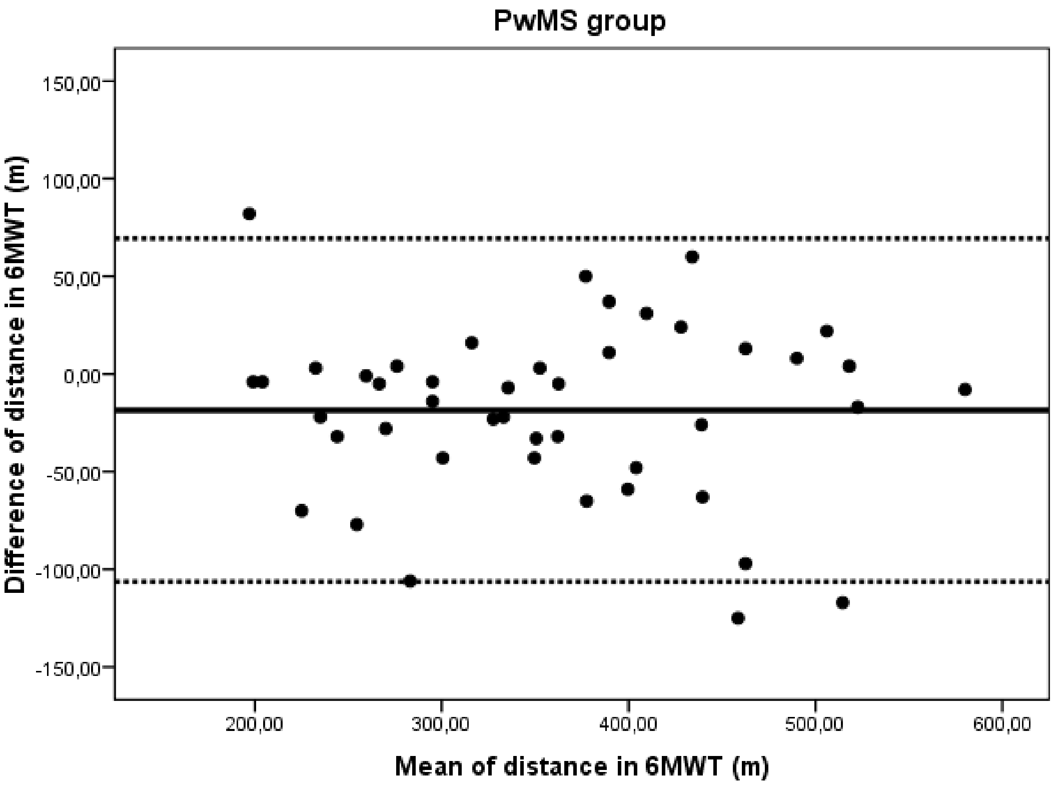
PwMS group
150,00
100,00
50,00
0,00
-50,00
-100,00
-150,00
Difference of distance in 6MWT (m)
200,00
300,00
400,00
500,00
600,00
Mean of distance in 6MWT (m)

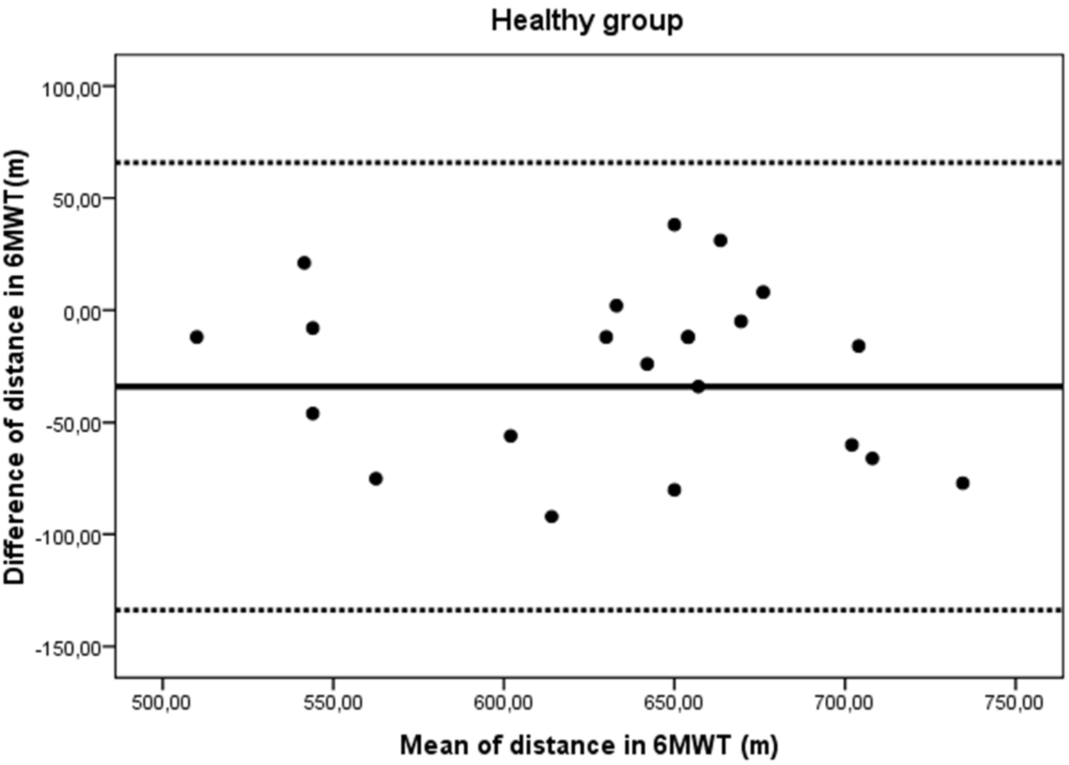
Healthy group
100,00
50,00
0,00
-50,00
-100,00
-150,00
Difference of distance in 6MWT(m)
500,00
550,00
600,00
650,00
700,00
750,00
Mean of distance in 6MWT (m)

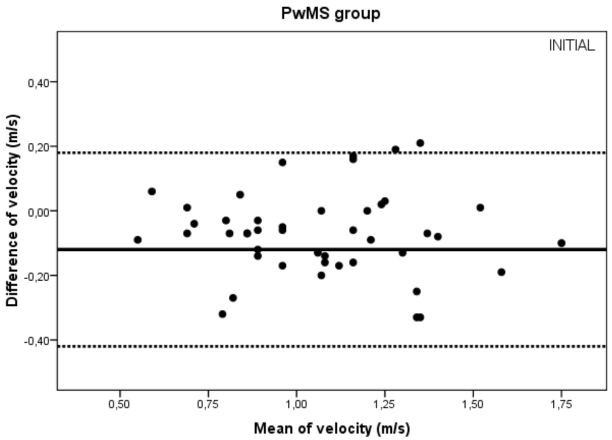
PwMS group
INITIAL
Difference of velocity (m/s)
Mean of velocity (m/s)

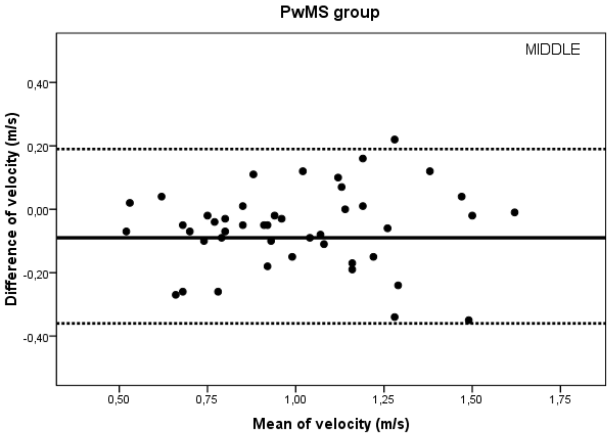
PwMS group
MIDDLE
Difference of velocity (m/s)
Mean of velocity (m/s)

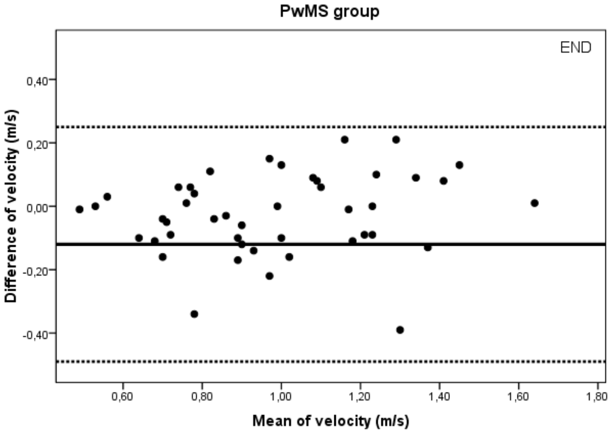
PwMS group
END
Difference of velocity (m/s)
Mean of velocity (m/s)

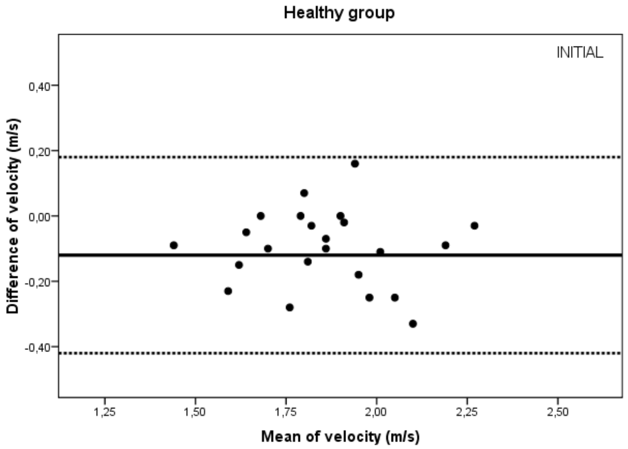
Healthy group
INITIAL
Difference of velocity (m/s)
Mean of velocity (m/s)

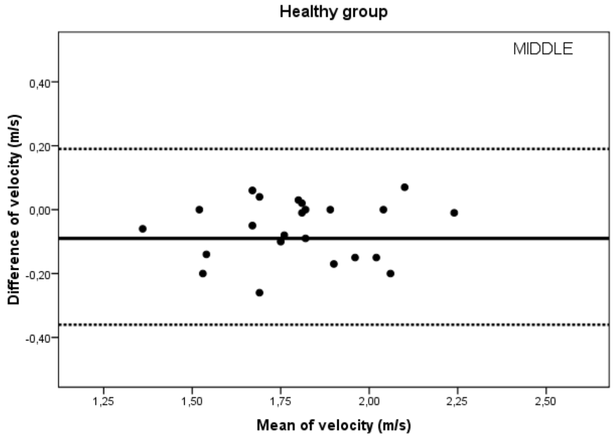
Healthy group
MIDDLE
Difference of velocity (m/s)
Mean of velocity (m/s)

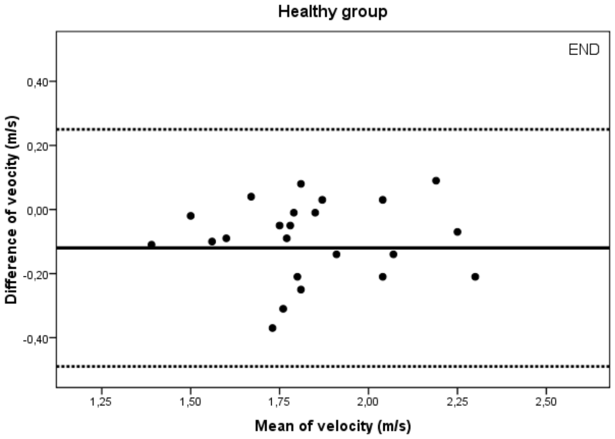
Healthy group
END
Difference of veocity (m/s)
Mean of velocity (m/s)

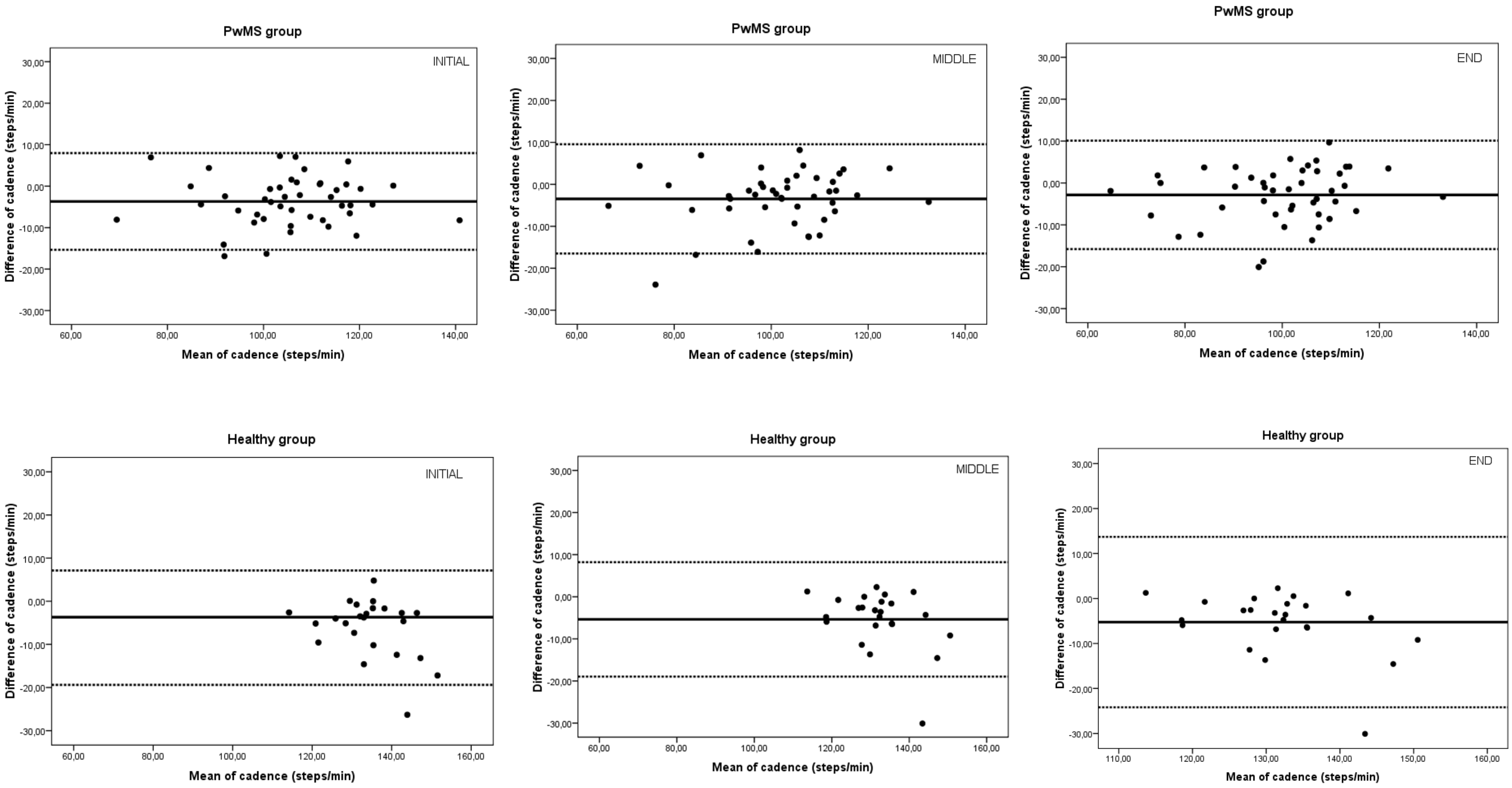
PwMS group
INITIAL
PwMS group
MIDDLE
PwMS group
END
Healthy group
INITIAL
Healthy group
MIDDLE
Healthy group
END
Difference of cadence (steps/min)
Mean of cadence (steps/min)

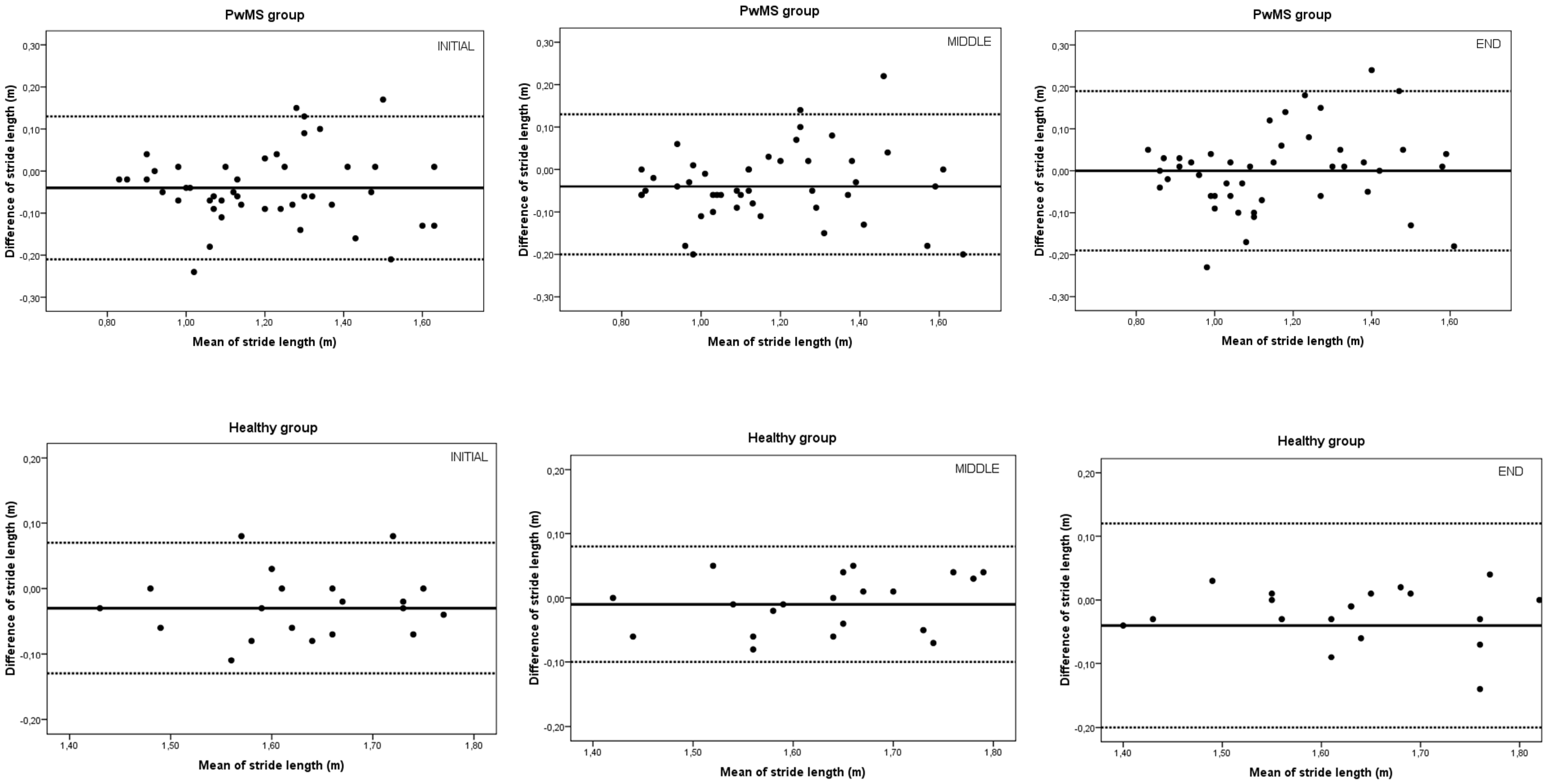
PwMS group
INITIAL
Difference of stride length (m)
Mean of stride length (m)
0,30
0,20
0,10
0,00
-0,10
-0,20
-0,30
0,80
1,00
1,20
1,40
1,60
PwMS group
MIDDLE
Difference of stride length (m)
Mean of stride length (m)
PwMS group
END
Difference of stride length (m)
Mean of stride length (m)
Healthy group
INITIAL
Difference of stride length (m)
Mean of stride length (m)
0,20
0,10
0,00
-0,10
-0,20
1,40
1,50
1,60
1,70
1,80
Healthy group
MIDDLE
Difference of stride length (m)
Mean of stride length (m)
Healthy group
END
Difference of stride length (m)
Mean of stride length (m)

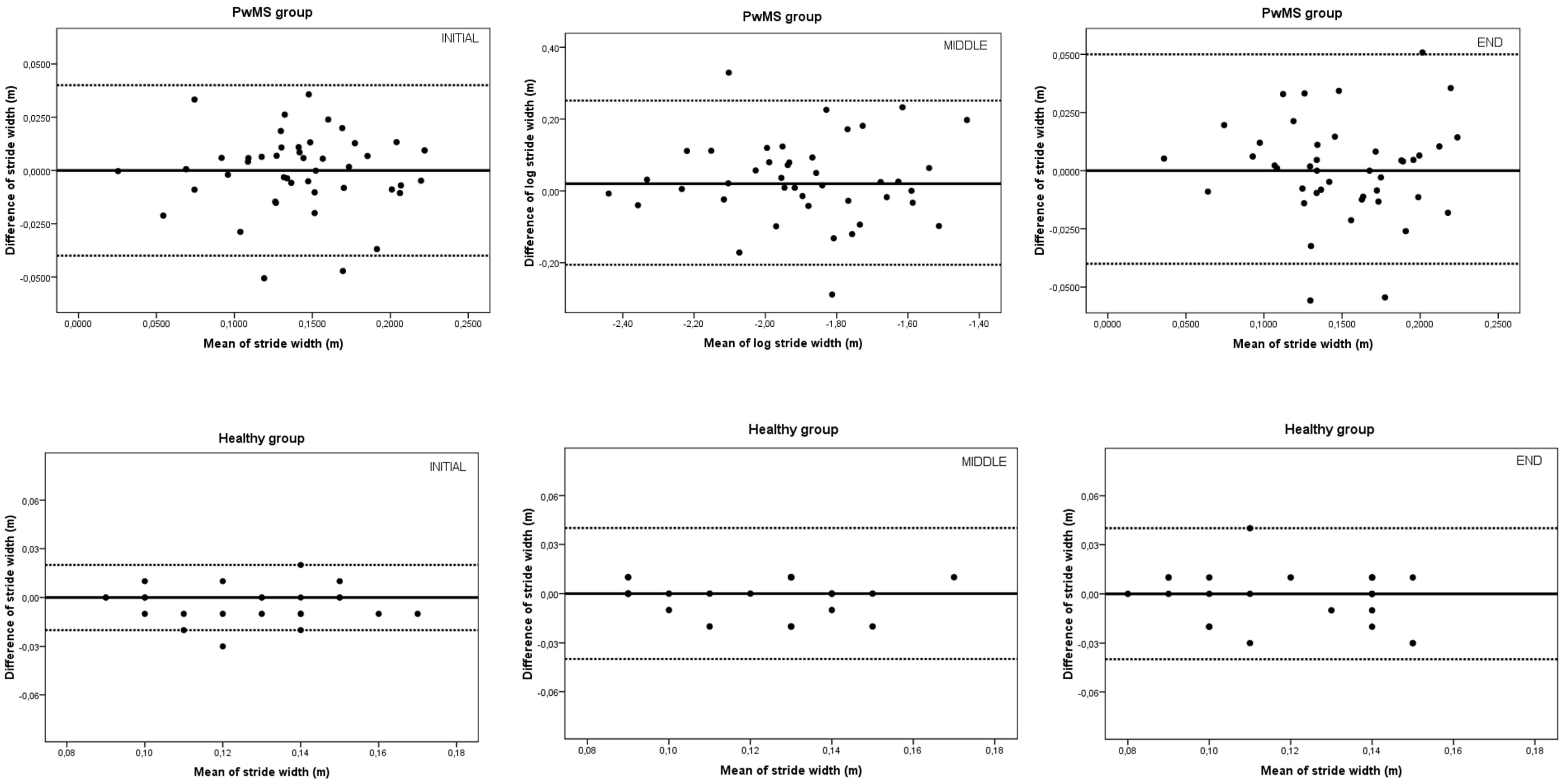
PwMS group
INITIAL
Difference of stride width (m)
Mean of stride width (m)
PwMS group
MIDDLE
Difference of log stride width (m)
Mean of log stride width (m)
PwMS group
END
Difference of stride width (m)
Mean of stride width (m)
Healthy group
INITIAL
Difference of stride width (m)
Mean of stride width (m)
Healthy group
MIDDLE
Difference of stride width (m)
Mean of stride width (m)
Healthy group
END
Difference of stride width (m)
Mean of stride width (m)

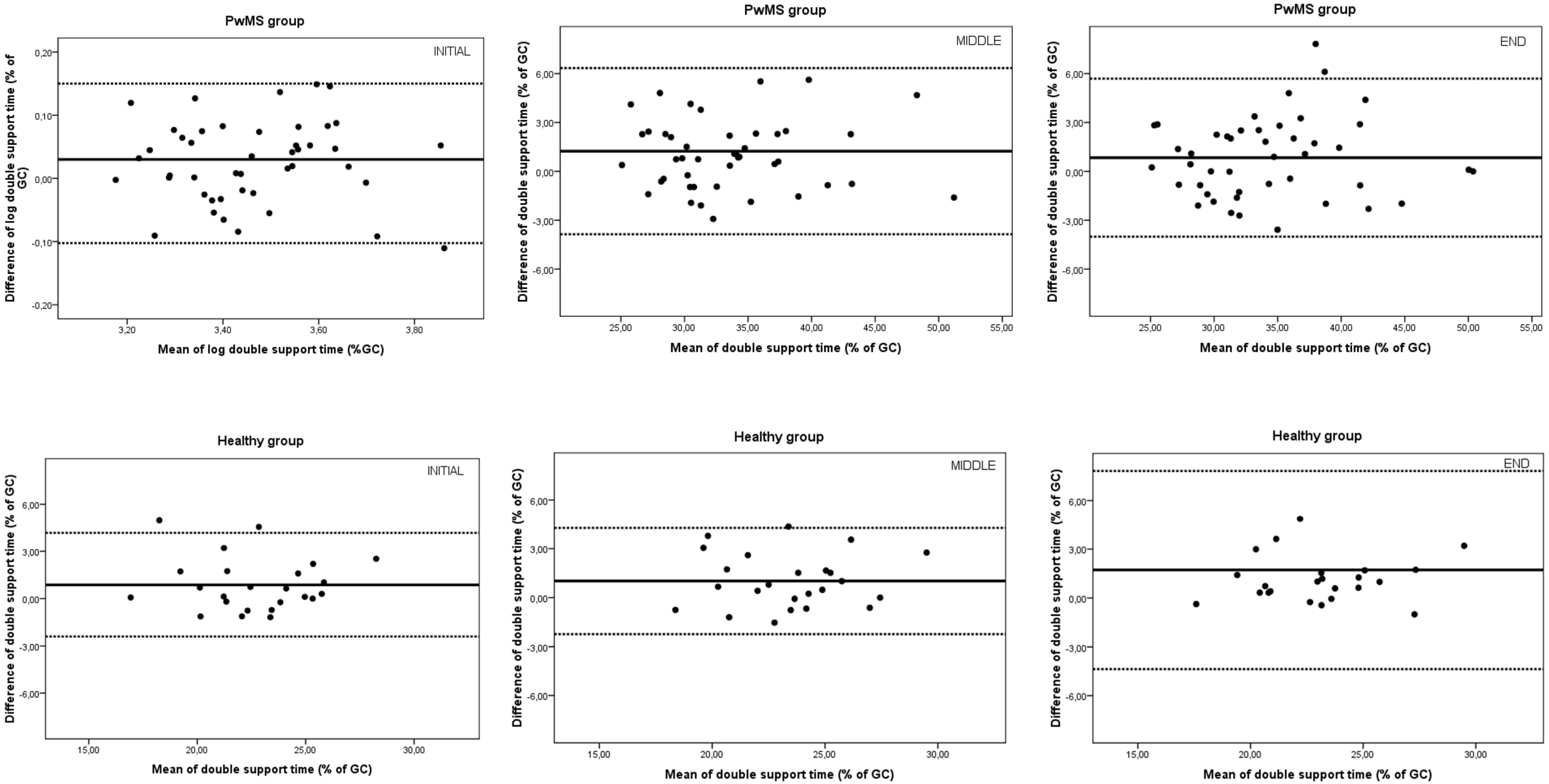

Figure 1. Bland Altman plot with 95% limits of agreement (LoA). Vertical axis: difference between test and retest values for distance in the 6-MWT and Spatio-temporal (ST) variables. Horizontal axis: mean of test and retest values for each variable studied. The black horizontal line represents the mean difference between test and retest (systematic bias). The dotted lines represent the limits of agreement. Abbreviations: 6-MWT, 6-Minute Walk Test; GC, Gait cycle.
